# Psychological distress and quality of life in older persons: relative contributions of fixed and modifiable risk factors

**DOI:** 10.1186/1471-244X-13-249

**Published:** 2013-10-08

**Authors:** Joanna Atkins, Sharon L Naismith, Georgina M Luscombe, Ian B Hickie

**Affiliations:** 1 Brain & Mind Research Institute, University of Sydney, Camperdown, NSW 2050, Australia; 2 School of Rural Health, Sydney Medical School, University of Sydney, Orange, Australia

**Keywords:** Older persons, Psychological distress, Quality of life, Risk factors

## Abstract

**Background:**

With a rapidly ageing population and increasing life expectancy, programs directed at improving the mental health and quality of life (QOL) of older persons are extremely important. This issue may be particularly relevant in the aged-care residential sector, where very high rates of depression and poor QOL are evident. This study aims to investigate the fixed and modifiable risk factors of psychological distress and QOL in a cohort of Australians aged 60 and over living in residential and community settings.

**Methods:**

The study examined the relationship between demographic, health and lifestyle factors and the outcome variables of self-reported QOL and psychological distress (K10 scores) based on data from 626 Australians aged 60 and over from the *45 and Up Study* dataset. Univariate and multivariate regression analyses (performed on a subset of 496) examined risk factors related to psychological distress and QOL adjusting for age and residential status.

**Results:**

Significant psychological distress was experienced by 15% of the residential sample and 7% of the community sample and in multivariate analyses was predicted by older age, more functional limitations, more time spent sleeping and lower levels of social support (accounting for 18% of the variance). Poorer QOL was predicted by more functional limitations and greater levels of psychological distress. Together these variables accounted for 35% of the variance in QOL ratings.

**Conclusions:**

While psychological distress was more common in residential settings, programs targeting modifiable risk factors have the potential to improve QOL and reduce psychological distress in older persons living in both residential and community settings. In particular, promoting health and mobility, optimising sleep-wake cycles and increasing social support may reduce levels of psychological distress and improve QOL.

## Background

With a rapidly ageing population and increasing life expectancy, programs directed at improving the mental health and quality of life (QOL) of older persons are extremely important. This issue may be particularly relevant in the aged-care residential sector, where very high rates of depression and poor QOL are evident [1]. Depression in older persons is also a public health problem, since it is associated with increased physical morbidity and mortality [2,3], decreased functional status [4], high health service utilisation [5] and increased rates of progression to dementia [6].

In order to devise optimal intervention programs that target both psychological distress and QOL in older people, it is necessary to consider both fixed and modifiable risk factors [7]. A number of fixed risk factors which have been shown to affect psychological distress in later life are age, gender and educational status [8]. In addition, a number of potentially modifiable risk factors have also been identified, for example, mental health [9], activity levels [10], social support [11,12], sleep [13], functional status [14], physical health burden [15] and alcohol consumption [16].

A strong relationship between quality of life and depression in the elderly has been noted in a number of studies [9,17]. For example, a study by Borowiak and Kostka [18] of 312 elderly persons found that depression was the strongest predictor of QOL in both community dwelling and institutionalised elderly.

Social support has been demonstrated to be important in both quality of life and depression. For example, a study of community dwelling older persons in Japan [19] found that the greater the number of friends and participation in social activities the less likely the person was to be depressed. In Ireland, a large scale study [11] of 1334 community dwelling adults aged 65 and over examined two domains of social support: a family domain (distance from and frequency of contact with relatives) and a social engagement domain (participation in social activities and contact with friends and neighbours). They found that the family domain was not associated with depression or quality of life measures but higher levels of social engagement were significantly associated with higher levels of quality of life and reduced prevalence of depression. In separate analyses, the same group of researchers [12] also found that loneliness accounted for 70% of depressed mood in their elderly sample.

The relationship between depression and functional impairment has been demonstrated by a number of researchers (see [14] for a review). For example, Eisses et al. in a study of older persons in nursing homes [20] found that functional impairment was the strongest risk factor for depression. In a prospective study of patients over 60 years, Callahan et al. [21] found that those who were depressed at baseline reported nearly twice the levels of functional impairment at follow up (average 45 months) than those without depressive symptoms at baseline.

A number of studies have demonstrated the association between quality of life and physical illness [15], indicating that the higher the medical burden the higher the risk for depression [22,23]. Studies have confirmed associations between depression and heart disease [24-26]; diabetes [27]; chronic obstructive pulmonary disease, bronchitis and asthma [28,29]; cancer [30] and arthritis [31].

The relationship between sleep problems and depression is well documented. Studies show that between 50-90% of people with depression have sleep disturbances [13,32]. Sleep disturbance is listed as one of the diagnostic criteria of depression in DSM-IV [33] and often precedes and predicts depression [34,35]. Livingstone et al. [36] found that the most significant predictor of future depression in older people was sleep disturbance. Perlis et al. [37] found that for older people with persistent insomnia there was a six fold risk of developing a first episode of depression.

Evidence suggests that physical activity levels are related to mental well-being in older people. For example, a number of studies have demonstrated the effectiveness of exercise programs in reducing depression in this age group [38-43]. A study by Lampinen et al. [44] that examined a large group of older persons prospectively over an eight year follow up period found that better well-being (including lower levels of depressive symptoms) at follow up was predicted indirectly by greater activity levels mediated through better mobility status and physical health at baseline. Another study by Strawbridge et al. [10] found that physical activity in the elderly protected them against depression over a five year follow up period. Fox et al. [45] found that the amount of daily activity (as measured by accelerometers) and the amount of time spent participating in moderate intensity physical activity were weakly related to quality of life, subjective well-being and depression. In addition sedentary time was also weakly negatively related to psychological health and well-being but not to depression specifically.

Prior studies that have assessed the contributions of fixed and modifiable risk factors to QOL and psychological distress in older person have generally been conducted in smaller sample sizes, have not considered QOL and psychological distress concurrently across older persons in both community and residential settings and have generally been in younger aged samples of older persons than the current study. This study aims to address this by using data from a large scale study, examining older persons in the community and aged care settings and examining psychological distress and QOL concurrently. The current study uses data from the Australian *45 and Up Study*[46]. This study is a population-based cohort study of health and well-being factors in the 45 and over age group and is the largest, most inclusive and recent epidemiological study of older persons in Australia. This large research project aims to provide a long-term collaborative resource in order to gather evidence to inform policy to support healthy ageing. It is within this context that the current study aimed to examine the links between fixed and modifiable risk factors for psychological distress and reduced QOL in an older Australian sample.

## Method

### Sample

**Figure 1:**
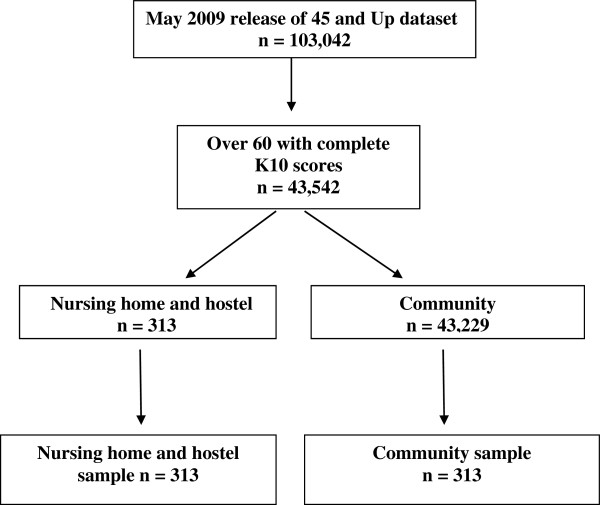
Sample selection flow chart.

Data from the *45 and Up Study* were utilised. This is a prospective self-report postal survey of persons from New South Wales aged 45 and over randomly selected from the Medicare Australian enrolment database. The study over-samples people over the age of 80 and people from rural areas (by a factor of two). The current study utilises a sub-sample of persons from the May 2009 release of the dataset (N = 103,042, data collected between June, 2004 and December, 2008). For the purposes of this study, an ‘older’ person was defined as someone 60 years and over. Inclusion criteria for the current study were being 60 years old or over, and having completed the measure of psychological distress – the Kessler Psychological Distress Scale (K10) [47,48]. Having dementia was not an exclusion criteria but it is likely that persons with this disorder would be self-selected out of the sample as they may have had difficulty completing the survey (the survey did not ask respondents if they had dementia). Of those meeting the inclusion criteria, there were 313 persons living in residential settings (108 in nursing homes and 205 in ‘hostels’). In Australia, nursing homes are generally for persons with high support needs and hostel accommodation for persons with low support needs. A comparison sample of 313 persons matched for age and gender were selected from the remaining respondents who met the inclusion criteria and lived in the community (see Figure 1).

### Measures

All of the variables examined in the present study were derived from the self-report *45 and Up* study questionnaire. Where the *45 and Up* variables included measures from validated scales they have been identified below.

### Outcome measures

#### Psychological distress

The K10 [49] is a well validated and utilised screening measure of psychological distress comprising ten questions on a 5-point scale ranging from ‘none of the time’ through to ‘all of the time’ and yields an additive score ranging from 10 through to 50. Questions relate to both depression and anxiety symptoms and combine to form the concept *psychological distress.* In addition to continuous scores, a binary variable was calculated for descriptive purposes using a cutoff score of ≥ 22. This cutoff has been used in a number of studies, for example the Australian Bureau of Statistics National Health Surveys (as described in [50]). Score ranges: 10–15 indicates low psychological distress, 16–21 = moderate, 22–29 = high, 30–50 = very high distress.

#### Quality of life

Self-rated QOL was based on one self report question: “In general, how would you rate your quality of life?”. This was scored on a Likert scale from 1 to 5 corresponding to ‘poor’, ‘fair’, ‘good’, ‘very good’ and ‘excellent’.

### Fixed and modifiable risk factors

#### Fixed risk factors

•*Demographic factors*: age, gender, usual annual household income (coded as < $30,000 per annum versus $30,000+ per annum) and residential status (nursing home/hostel/community).

#### Potentially modifiable risk factors

•*Functional limitations:* These were assessed using the *Physical Functioning subscale* of the *Medical Outcomes Study 36-item Short-Form Health Survey (SF-36)*[51]. This subscale has ten questions that examine limitations in usual role activities because of physical health problems. Activities include: lifting or carrying shopping, climbing stairs, walking, bending, stooping, kneeling, bathing and dressing. The Rand scoring method was applied [52], where all ten questions are scored on a scale from 0 to 100 (100 representing the highest level of functioning possible), and an average of the ten question scores is then calculated.

•*Physical health burden*: A health burden score was calculated based on the number of five major illnesses (cancer, cardiovascular disease, diabetes, stroke and Parkinson’s disease) endorsed by the survey respondent, whereby a history of a specific illness (“has the doctor ever told you that you have…”) contributed a score of one, yielding a final score out of five. This method of calculating physical health burden is similar to that used in other studies [53-55].

•*Activity level:* Three questions from the *Active Australia Survey* (Australian Institute of Health and Welfare, 2003) were included. These examine the amount of time spent each week (minutes) ‘walking’ and participating in ‘moderate’ and ‘vigorous’ activities. In accordance with the *Active Australia Survey* scoring criteria, minutes spent in ‘vigorous’ activity are doubled. A final score is derived by adding the scores for ‘walking’, ‘moderate’ and ‘vigorous’ activity together.

•*Hours of sleep*: Participants were asked to assess: “about how many hours in each 24 hour day you usually spend sleeping (including at night and naps)?”.

•*Time spent outdoors*: Participants were asked: “about how many hours a day would you usually spend outdoors on a weekday” and “about how many hours a day would you usually spend outdoors on the weekend?” These measures were then totalled to give hours per week.

•*Social support*: Four questions from The *Duke Social Support Index*[56] were included. Three questions examined the number of times in the last week that time was spent with family and friends, talking on the phone with family or friends and taking part in group activities. The fourth question assessed the number of people outside of the family and within one hour of home that can be depended on. All items were recoded according to the guidelines in the *Duke Social Support* manual to give a score between 1 and 3 and are then summed to give a total score out of 12.

•*Alcohol consumption*: Participants were asked to assess the number of alcoholic drinks they consume each week.

•*Time spent sitting*: Participants were asked: “about how many hours in each 24 hour day do you usually spend sitting?”

•*Psychological distress:* Symptoms of psychological distress over the last four weeks were measured by the K10 (as described above).

In addition, participants were asked if their doctor had ever told them they had depression, and if they had been treated for depression in the past month; and if the doctor had ever told them they had anxiety and if they had been treated for anxiety in the past month.

### Statistical analysis

Analyses were conducted using SPSS Version 19. Non parametric tests were used to examine differences between hostel, nursing home and community samples on continuous data as all these variables had skewed distributions. The Kruskal Wallis test was used for three group comparisons of continuous or ordinal variables and the Mann–Whitney *U* test for the two group analyses (nursing home vs hostel; nursing home vs community; hostel vs community; Z statistic reported). Bonferroni corrections were used in the two group analyses. Chi squared analyses were used for assessing relationships among categorical variables. The ordinal variable QOL was dichotomised into the categorical variable *poor/fair* versus *good/very good/excellent*. Correlations between K10 and potential predictor variables were examined using Spearman’s rho correlation coefficients (r_s_) and univariate binary logistic regression analyses were used to determine the potential predictors for QOL. Multiple regression analyses were used to determine the relative contribution of fixed and modifiable risk factors for the outcome variables of K10 and QOL. Multiple linear regression models were used for K10 with forced entry of fixed risk factors (block 1, age, residential status; method = enter), followed by modifiable risk factors (block 2; method = stepwise). For missing data cases were excluded pairwise. We also ascertained the semipartial (part) correlation associated with each variable after controlling for other predictors, in order to determine the unique variance associated with that predictor. Logistic regression models were used to ascertain predictors for QOL, with forced entry of fixed risk factors (block one) and modifiable risk factors (block 2). Where appropriate, analyses used two tailed tests and the significance level was set at 0.05.

#### Data transformations

The following modifiable risk factors were curtailed for extreme outliers: Activity level (capped at 1680 minutes per week in accordance with instructions in the Active Australia Survey manual), hours of sleep (capped at 15 hours per day), time spent outdoors (capped at 70 hours per week), alcohol consumption (capped at 36 drinks per week), and time spent sitting (capped at 16 hours per day).

## Ethics approval

The study used de-identified data from the *45 and Up* study database where participants had given informed consent for their data to be used for research purposes as approved by the *45 and Up* dataset owners.

## Results

Table 1 shows demographic, health and lifestyle factors for the three groups of participants.

**Table 1:** Demographic, health and lifestyle factors for nursing home (n = 108), hostel (n = 205) and community (n = 313) sample of elderly persons

	**Residential status**			**Group comparison**	**Multiple comparisons**		
	**Nursing home (NH)**	**Hostel (H)**	**Community (C)**		**NH *vs *H Z**	**NH *vs *C Z**	**H *vs *C Z**
Gender (female) % (n)	57.4 (62)	58.0 (119)	57.8 (181)	0.0			
Income (< $30,000 per annum) % (n)	83.1 (59)	75.0 (87)	70.8 (165)	4.3			
QOL scores (good/v. good/excellent) % (n)	56.3 (58)	61.2 (115)	83.8 (244)	43.4**	0.7	32.2^b^	31.3^b^
K10 score (10–50) med (IQR)	14.0 (9.0)	14.0 (7.0)	12.0 (4.0)	29.1**	−0.02	−3.7^b^	−4.9^b^
Age (years) med (IQR)	83.2 (14.9)	82.2 (12.6)	80.8 (4.9)	8.6*	−4.5	−2.1	−2.6^a^
Physical health burden (0–5) med (IQR)	1.0 (2.0)	1.0 (1.0)	1.0 (1.0)	6.7*	−1.1	−0.9	−2.6^a^
Functional limitations (0–100) med (IQR)	10.0 (68.3)	30.0 (50.0)	70.0 (55.0)	91.9**	−2.9^b^	−7.2^b^	−8.1^b^
Physical activity (mins per week) med (IQR)	60.0 (420.0)	150.0 (495.0)	270.0 (629.0)	32.2**	−2.0	−5.1	−4.1^b^
Sleep (hours per day) med (IQR)	9.0 (5.0)	8.0 (3.0)	8.0 (2.0)	12.4**	−1.5	−3.2^b^	−2.4^a^
Time outdoors (hours per week) med (IQR)	7.0 (20.0)	9.0 (18.8)	16.0 (19.0)	45.9**	−2.0	−5.8^b^	−5.1^b^
Social support (scaled scores) med (IQR)	8.0 (3.0)	8.0 (2.5)	9.0 (2.0)	27.6**	−0.2	−3.6^b^	−4.8^b^
Alcohol (drinks per week) med (IQR)	0 (3.8)	0 (3.0)	2.0 (7.0)	21.5**	−0.2	−3.3^b^	−4.1^b^
Sitting (hours per day) med (IQR)	8.0 (7.0)	7.0 (6.0)	5.0 (3.0)	34.3**	−1.4	−5.4^b^	−4.1^b^

Note: Kruskal Wallis non-parametric tests used due to skewed and/or unequal distributions, *p < 0.05, **p < 0.01; Multiple comparisons for continuous/ordinal data conducted with Mann Whitney U, Test (Z statistic reported), with a Bonferroni correction to the alpha level of 0.05/3 (0.017) and for categorical data conducted with Chi Squared. ^a^ denotes p < 0.017, ^b^ denotes p <0.01.

NH, nursing home; H, hostel; C, community; med, median; IQR, Interquartile Range.

### Fixed risk factors

The median age of the entire sample was 81.2 years (IQR = 8.3) and 57.8% were female. The majority of the sample had an income of less than $30,000 per annum with no significant differences between groups.

As the only significant difference between the nursing home and hostel samples was for functional limitations (Z = −2.9, p = 0.003), the hostel and nursing home samples have been combined and compared with the community sample in subsequent analyses.

### a) Comparison of residential and community samples

#### Psychological distress and QOL

The community group had significantly lower levels of psychological distress (K10 scores: Z = −5.4, p < 0.001) and higher QOL scores (χ^2^ = 42.6, df = 1, p < 0.001) than the residential group. Overall, 11% of the total sample had K10 scores ≥ 22 indicating significant psychological distress (15.3% of the residential sample and 6.7% of the community sample). This difference was significant (χ^2^ = 11.9, df = 1, p = 0.001). Overall, the median K10 scores were 13.0 (IQR = 5.0) for the residential and 13.6 (SD = 5.1) for the community sample, indicating psychological distress in the low range for both groups. For QOL, 83.8% of the community group rated their QOL as *good*, *very good* or *excellent* compared to 59.5% of the residential group.

#### Self-reported experience of depression and anxiety

For the residential sample, 25.3% of those who answered the question (n = 59/233), reported having been told by their doctor at some time in their lives that they had depression, compared to 4.9% of the community sample (n = 11/224) (χ^2^ = 36.7, df = 1, p < 0.001). For the residential sample, 17.2% (n = 40/233) said they had been treated for depression in the last month compared to 3.1% (n = 7/224) of the community sample (χ^2^ = 24.4, df = 1, p < 0.001). In relation to anxiety, 15.0% (n = 35/233) of the residential sample, who answered the question, reported having been told by their doctor at some time in their lives that they had anxiety compared to none (n = 0/313) of the community sample (χ^2^ = 50.2, df = 1, p < 0.001) and 9.9% (n = 23/233) of the residential sample reported having been treated for anxiety in the last month compared to none (n = 0/313) of the community sample (χ^2^ = 32.3, df = 1, p < 0.001).

#### Potentially modifiable risk factors

The community group had lower levels of physical health burden (Z = −2.3, p = 0.020) than the residential group. The community sample spent, on average, significantly more time outdoors than the residential sample (Z = −6.5, p < 0.001), had significantly fewer functional limitations (Z = −9.4, p < 0.001) and engaged in significantly more activity (Z = −5.4, p < 0.001). They also reported consuming a significantly higher quantity of alcohol than the residential group (Z = −4.6, p < 0.001), had significantly more social support (Z = −5.3, p < 0.001), had shorter sleep durations (Z = −3.2, p = 0.001), and spent less time sitting (Z = −5.6, p < 0.001).

### b) Predictors of psychological distress and quality of life

In order to determine the most pertinent predictors of psychological distress and QOL, bivariate analyses were firstly conducted. Table 2 displays the univariate associations between K10 scores and fixed and modifiable risks for continuous variables and Table 3 the univariate associations between these factors and QOL. The data highlight the significant degree of inter-relationships between these variables. In addition to the data shown in Table 2, gender was not a significant univariate predictor.

**Table 2:** Univariate association between K10 scores and risk factors (n = 626)

	**K10**
	**r**_**s**_
**Age**	0.089*
**Sleep** (hours per day)	0.175**
**Social support** (scaled scores)	−0.213**
**Activity** (minutes per week)	−0.325**
**Time spent outdoors** (hours per week)	−0.254**
**Alcoholic drinks** (number per week)	−0.134**
**Time spent sitting** (hours per day)	0.143**
**Physical health burden** (0–5)	0.163**
**Functional limitations** (0–100)	−0.430**

K10 calculations used Spearmans rho (r_s_).

** Correlation is significant at the 0.01 level.

* Correlation is significant at the 0.05 level.

**Table 3:** Univariate logistic regression for QOL

	**Odds ratio**	**95% CI**	**P**
**Age**	0.951	0.927–0.975	< 0.001
**Residential status**^**†**^	0.282	0.191–0.417	< 0.001
**Activity** (minutes per week)	1.002	1.001–1.003	< 0.001
**Physical health burden** (0–5)	0.685	0.566–0.830	< 0.001
**Functional limitations** (0–100)	1.036	1.029–1.044	< 0.001
**Time spent outdoors** (hours per week)	1.059	1.039–1.078	< 0.001
**Social support** (scaled scores)	1.349	1.207–1.509	< 0.001
**K10** (10–50)	0.815	0.780–0.852	< 0.001
**Sleep** (hours per day)	0.837	0.762–0.919	< 0.001
**Alcoholic drinks** (number per week)	1.069	1.029–1.110	0.001
**Time spent sitting** (hours per day)	0.885	0.840–0.933	< 0.001

^†^ Residential status is a categorical variable: reference category = Community.

#### Multivariate analyses

Activity levels and income were not included in the multivariate analyses because of a large amount of missing data giving a final sample size of 496 (residential sample = 276, community sample = 220) for the multivariate analyses. Those who were included in the multivariate analyses were compared with those who were not included on the variables of age, income, gender and residential status. There were no significant differences for age or income but the gender ratio was altered significantly from 42.2% male/57.8% female to 36.3% male/63.7% female and the ratio of residential/community changed from 50.0%/50.0% to 55.6%/44.4%. It should be noted however that gender was not a significant univariate predictor and was not included in the multivariate analyses.

#### Multivariate analyses for psychological distress

Table 4 shows the results of the multiple regression model for predictors of psychological distress (K10). After forced entry of fixed risks (age and residential status), the modifiable risks shown to have a significant association in univariate analyses (sleep, social support, time spent outdoors, alcohol consumption, time spent sitting, physical health burden, and functional limitations) were subjected to stepwise elimination. Of the fixed risks, age remained significant, uniquely accounting for 0.9% of the variance. Of the modifiable risks, social support remained significant, uniquely accounting for 1.8% of the variance, functional limitations uniquely accounted for 8.1% of the variance and sleep uniquely accounted for 0.7% of the variance. Overall, these variables accounted for 17.6% of the variance in K10 scores (F (df = 5, 490) = 21.0, p < 0.001).

**Table 4:** Multiple Linear Regression of fixed and modifiable risk factors on K10 scores (n = 496)

**Adjusted for age and residential status**				
	**Beta weight**	**t**	**Unique r**^**2**^	**p**
**Fixed risk factors (block 1)**				
*Forced entry variables*				
Age	−0.105	−2.356	0.9	0.019
Residential status	−0.015	−0.334		0.809
**Modifiable risk factors (block 2)**				
*Included variables*				
Functional limitations	−0.347	−7.142	8.1	< 0.001
Sleep	0.089	2.066	0.7	0.039
Total social support	−0.142	−3.309	1.8	0.001
*Excluded variables*				
Physical health burden, Time spent outdoors, Alcoholic drinks, Time spent sitting				
	**Total R**^**2**^	**F (df)**	**p**	
	17.6	21.0 (5,490)	< 0.001	

#### Multivariate analyses for quality of life

As shown in Table 5, after controlling for fixed risks (age and residential status), modifiable variables shown to have a significant association in univariate analyses (sleep, social support, time spent outdoors, alcohol consumption, time spent sitting, physical health burden, functional limitations and K10) were entered into a separate block. Neither of the fixed risks were significant predictors of QOL. Of the modifiable risks, only psychological distress and functional limitations remained significant predictors. Overall, these variables accounted for 34.8% of the variance in QOL scores.

**Table 5:** Multiple logistic regression of fixed and modifiable risk factors on quality of life (n = 496)

**Adjusted for age and residential status**			
	**Odds ratio**	**95% CI**	**P**
**Fixed risk factors (block 1)**			
Age	1.005	0.966–1.046	0.809
Residential status^†^	0.087	0.302–1.084	0.572
**Modifiable risk factors (block 2)**			
Physical health burden	0.821	0.605–1.113	0.204
Functional limitations	1.025	1.013–1.037	< 0.001
Time spent outdoors	1.017	0.997–1.038	0.100
Social support	0.989	0.835–1.170	0.894
K10	0.817	0.768–0.870	< 0.001
Sleep	0.961	0.835–1.106	0.581
Alcoholic drinks	1.028	0.975–1.085	0.306
Time spent sitting	1.030	0.949–1.118	0.479
	**Total R**^**2**^		
	34.8		

^†^ Residential status is a categorical variable: reference category = Community.

## Discussion

This study examined the relative contributions of fixed and potentially modifiable risk factors for psychological distress and QOL in a combined sample of residential and community dwelling persons aged 60 and over. While psychological distress was found to be higher, and QOL lower in the residential sample, a number of risk factors were found to be associated with psychological distress and QOL for both community and residential samples. The main factor associated with higher psychological distress was poorer functional status, accounting for around 8% of the variance. Others factors that made smaller but significant contributions were reduced social support and more time spent sleeping. Variables that were found to be associated with lower QOL included higher levels of functional impairment and higher psychological distress.

The results of the current analyses confirm findings of previous studies which indicate significantly higher levels of psychological distress in residential populations compared to those for community dwelling older persons [1].

The current research has identified a number of risk factors for psychological distress and QOL. Awareness of these risk factors is important so that appropriate prevention and treatment interventions can be developed. Modification of these risk factors could have an important impact on successful ageing. Some risk factors lend themselves more readily to intervention than others. For example, even after controlling for fixed risk factors, social support was a risk factor for psychological distress uniquely accounting for 1.8% of the variance. While this amount is small, it is still significant and worthwhile attempting interventions to improve social support. Intervention programs in residential care might include volunteer visitor programs, group and social activity programs and the employment of recreation officers. Initiatives promoting outings and visits by family and friends could also be specifically targeted. Other non-pharmacological interventions for older people with psychological distress may also be helpful, particularly problem-solving therapy, which may be most helpful for those in community settings [57]. The other variable that appeared to contribute significantly to psychological distress was sleep, whereby longer sleep periods were associated with higher psychological distress. In this regard, it is important to note that the mean sleep levels of this sample were longer than generally expected for older people (see [58] for a review). While the contribution of sleep was small – less than 1% - it is still worthwhile finding ways to improve sleep in older persons. While some studies suggest that promoting optimal circadian rhythms and sleep-wake functioning in older persons is advantageous for mood, further research exploring pharmacological and non-pharmacological interventions for both insomnia and hypersomnia are required (See review by [59]).

Psychological distress was an important contributor to QOL suggesting that ways of reducing psychological distress, such as improving social support and sleep are also likely to impact QOL. Improved recognition and treatment of depression and anxiety symptoms is another way that psychological distress could be reduced in older persons potentially leading to improved QOL. For example, evidence suggests that depression can be successfully treated in older adults [9] and that treatment leads to improvements in QOL [60], health status [61] and functional status [54]. It is important to educate older persons, their families and carers on the importance of maintaining social networks, treating depression, being physically active, spending time outdoors and maintaining health and mobility.

In the present study, functional limitations were considered as a potentially modifiable risk factor for both psychological distress and QOL. Exercise and physiotherapy programs may help to improve or maintain functional status, as well as improve mood. Physical health burden was also considered as a potentially modifiable risk factor and has been shown in a number of studies to have a strong relationship with depression [62,63]. In the current study, physical health burden did not predict psychological distress or QOL, however, it is important that older persons receive optimal treatment of physical illness to reduce both physical suffering and improve their QOL. In future public health planning, it is important to adopt an early intervention approach to promoting healthy ageing and in this regard targeting people in middle age is likely to be optimal [7].

There are a number of limitations to the current research. For example, QOL was assessed by only one question. The authors were restricted to the use of the questions used in the *45 and Up* study questionnaire. An empirically validated QOL scale would be a preferable method of assessing this construct. The current study relies on self-report data which may be unreliable and ideally should be compared with objective measures where possible. The data collected are cross-sectional so temporal relationships between the constructs cannot be assessed. There is evidence to suggest that those with poorer mental [64] and physical health [65] are less likely to participate in surveys which may mean that they are under-represented in the current analyses. In addition, the response rate for the *45 and Up* survey was only 18% which may limit its representativeness, though in this regard, it is noted that the over 80’s were over-sampled. Empirical work has been conducted by the *45 and Up* Study authors [66] comparing the results of their survey with results of the ‘New South Wales Population Health Survey’ [67], which is a study using computer assisted telephone interviews on the same population (asking similar questions) and with a response rate of 60%. They found almost identical results to questions with the same wording and conclude that high response rates are not necessary for generalisable results from cohort studies such as the *45 and Up* study. There are also a number of potentially modifiable risk factors that were unable to be analysed in this study. For example, environmental factors may be important and in this regard, considerable efforts have been devoted to the architecture and environment of aged care facilities. Cognitive activity may also be integral for optimising healthy brain ageing but these data were not collected either [68].

Future research is needed to implement and assess the most effective intervention strategies for improving modifiable risk factors for psychological distress and poor QOL in older persons. In particular, promoting health and mobility and increasing social support may be helpful. Any effects on depressive symptoms may, in turn, also optimise cognition and may even slow rates of progression to dementia [69].

## Conclusions

In this sample of older Australians, where the majority were aged 80 or above, a large proportion enjoyed good psychological health and QOL. While psychological distress was more common in residential settings, programs targeting modifiable risk factors have the potential to improve QOL and reduce psychological distress in older persons living in both residential and community settings. In particular, promoting health and mobility, optimising sleep-wake cycles and increasing social support may reduce levels of psychological distress and improve QOL.

## Competing interests

The authors declare that they have no competing interests.

## Authors’ contributions

JA performed the statistical analyses and wrote the paper. SLN supervised the study and assisted with revision of the paper. GML advised on statistical analysis of the data and assisted with revision of the paper. IBH supervised the research and assisted with revision of the paper. All authors read and approved the final manuscript.

## Pre-publication history

The pre-publication history for this paper can be accessed here:

http://www.biomedcentral.com/1471-244X/13/249/prepub
